# Sacrificial Cu Layer Mediated the Formation of an Active and Stable Supported Iridium Oxygen Evolution Reaction Electrocatalyst

**DOI:** 10.1021/acscatal.1c02968

**Published:** 2021-09-28

**Authors:** Anja Lončar, Daniel Escalera-López, Francisco Ruiz-Zepeda, Armin Hrnjić, Martin Šala, Primož Jovanovič, Marjan Bele, Serhiy Cherevko, Nejc Hodnik

**Affiliations:** † Department of Materials Chemistry, National Institute of Chemistry, Hajdrihova 19, 1000 Ljubljana, Slovenia; ‡ University of Nova Gorica, Vipavska 13, 5000 Nova Gorica, Slovenia; § Helmholtz-Institute Erlangen−Nürnberg for Renewable Energy, Forschungszentrum Jülich, Egerlandstrasse 3, 91058 Erlangen, Germany; ∥ Department of Analytical Chemistry, National Institute of Chemistry, Hajdrihova 19, 1000 Ljubljana, Slovenia

**Keywords:** iridium nanoparticles, oxygen evolution reaction (OER), titanium oxynitride (TiON) support, identical location transmission electron microscopy (IL-TEM), S-number

## Abstract

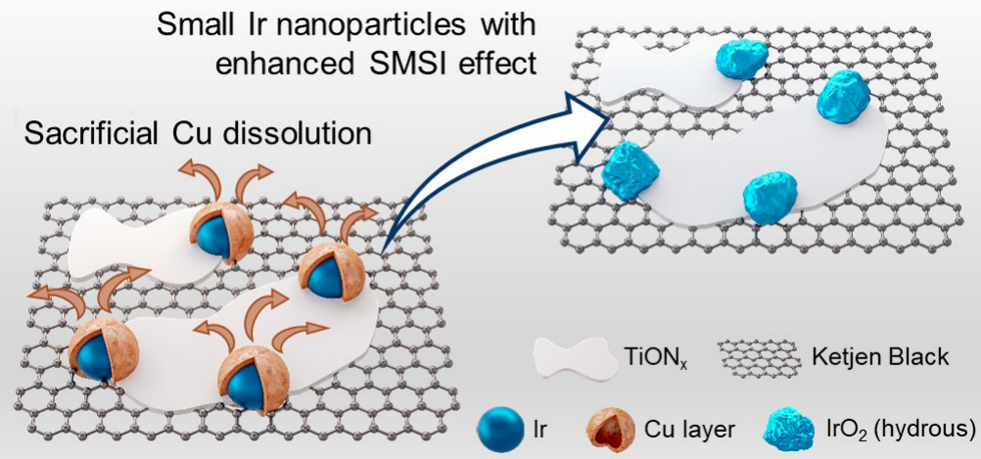

The production of hydrogen via a proton-exchange membrane water electrolyzer (PEM-WE) is directly dependent on the rational design of electrocatalysts for the anodic oxygen evolution reaction (OER), which is the bottleneck of the process. Here, we present a smart design strategy for enhancing Ir utilization and stabilization. We showcase it on a catalyst, where Ir nanoparticles are efficiently anchored on a conductive support titanium oxynitride (TiON*_x_*) dispersed over carbon-based Ketjen Black and covered by a thin layer of copper (Ir/CuTiON*_x_*/C), which gets removed in the preconditioning step. Electrochemical OER activity, stability, and structural changes were compared to the Ir-based catalyst, where Ir nanoparticles without Cu are deposited on the same support (Ir/TiON*_x_*/C). To study the effect of the sacrificial less-noble metal layer on the catalytic performance of the synthesized material, characterization methods, namely X-ray powder diffraction, X-ray photoemission spectroscopy, and identical location transmission electron microscopy were employed and complemented with scanning flow cell coupled to an inductively coupled plasma mass spectrometer, which allowed studying the online dissolution during the catalytic reaction. Utilization of these advanced methods revealed that the sacrificial Cu layer positively affects both Ir OER mass activity and its durability, which was assessed via S-number, a recently reported stability metric. Improved activity of Cu analogue was ascribed to the higher surface area of smaller Ir nanoparticles, which are better stabilized through a strong metal–support interaction (SMSI) effect.

## Introduction

The transition to a clean and sustainable society is predicted to occur by the use of green hydrogen as an energy vector that can replace fossil fuels.^1^ Proton-exchange membrane water electrolysis (PEM-WE) coupled with solar and wind electricity is considered one of the promising technologies for the production of “zero carbon emission” hydrogen, enabling intermittent renewable energy storage.^2,3^ However, due to the extremely corrosive environment in the PEM electrolyzer, the choice of catalyst materials is limited to platinum group metals (PGMs).^4^ The oxygen evolution reaction (OER), one of the half-reactions, is considered to be the bottleneck of the technology, as its sluggish kinetics contributes to the majority of the overpotential losses of the water-splitting process.^5^ An efficient catalyst would therefore be required to sufficiently increase the reaction rate of a complex four-electron process on the anode side, i.e., the OER. Iridium and its oxides are most often used as OER catalysts, as they present the best trade-off between activity and stability.^6,7^ However, for efficient electrochemical conversion, high loadings of this very rare metal are required.^8^ Even though the costs of the expensive catalysts in relatively small systems present less than 10% of the total cost, loadings of iridium will need to be reduced from approximately 2 mg_Ir_ cm^–2^ currently used to only 0.05 mg_Ir_ cm^–2^ for the TW scale-up of the technology, not only because of the price of noble metals but mostly due to the future supply constraints.^8,9^ Therefore, the development of new catalyst materials for the OER is demanded. As iridium is currently not replaceable, its utilization needs to be optimized. It can be achieved by increasing its surface area by decreasing the size, adjusting the morphology or crystallinity of the nanoparticles,^10−12^ by the formation of more active electrochemical amorphous oxide,^13^ development of a core–shell structure,^14^ or mixing iridium with different metals, such as Ru,^15^ Ni,^16^ Co,^17^ and Cu^18^ to tune its electrocatalytic performance. A further increase in the utilization of Ir nanostructures can also be achieved by supporting them onto the stable and conductive oxide supports. In commercial electrolyzers, TiO_2_ is often used as the support given that it is affordable and stable. However, due to the lack of conductivity, still relatively high iridium loadings are needed.^19^ As potential alternative supports, tin oxide^20^ and its doped analogues, such as antimony-doped tin oxide (ATO),^21−23^ indium-doped tin oxide (ITO),^24^ fluorine-doped tin oxide (FTO),^25^ and tantalum-doped tin oxide (TaTO)^26^ are often proposed; however, their use is still limited to the laboratory scale due to their stability constraints.^20,25^ One of the potential supports is also doped titanium oxides.^27−29^ In our recent reports,^28−30^ iridium nanoparticles were supported on a titanium oxynitride, TiON*_x_*, which was shown to have both proper electrical conductivity^28^ and electrochemical stability.^30^ Improved electrocatalytic properties of supported iridium are due to the fine dispersion of small iridium nanoparticles and SMSI (strong metal–support interaction) effect, which occurs between oxides and the supported Ir nanoparticles.^21,31,32^ As a result of the formation of a very thin layer of TiO_2_ on the surface of the support, its stability is improved while iridium nanoparticles still stay efficiently embedded and electrically wired. Density functional theory (DFT) calculations have also shown that the presence of nitrogen atoms additionally enhances the OER durability, as it reduces the tendency of the iridium nanoparticles to grow and thus contributes to the SMSI effect.^30^

Here, we present an improved material, where Ir nanoparticles are covered by a thin layer of Cu via a novel synthesis method: Ir/CuTiON*_x_*/C. Compared to the non-Cu analogue with the same Ir loading (Ir/TiON*_x_*/C), particle size distribution, and TiON*_x_*/C support, we observe a 35% boost in OER performance. The two materials were characterized by powder X-ray diffraction (XRD), X-ray photoelectron spectroscopy (XPS), and scanning transmission electron microscopy (STEM). Importantly, advanced electrochemical characterization was performed, namely, dissolution measurements performed by a scanning flow cell (SFC) coupled to the inductively coupled plasma mass spectrometer (ICP-MS) and local atomically resolved structural changes probed by identical location TEM (IL-TEM). We reveal that the addition of Cu increases Ir utilization and thus the electrocatalytic surface area, which directly impacts the OER activity of iridium nanoparticles. In addition, inherent iridium stability is also improved due to the enhanced SMSI effect of smaller Ir nanoparticles.

## Experimental Section

### Synthesis

Both samples, Ir/TiON*_x_*/C and Ir/CuTiON*_x_*/C, were prepared following the same protocol. In the first step of the synthesis, Ketjen Black EC-600JD (AkzoNobel) was first mixed with Ti-isopropoxide (Aldrich, 97%) and isopropanol (Honeywell, 99.8%) and then water (Milli-Q water 18.2MΩ.cm) was added to the obtained paste. After the addition of water, the mixture was dried for 1 h at 80 °C. In the case of Ir/CuTiON*_x_*/C, a 10 mL water solution of Cu(NO_3_)_2_·2.5H_2_O (Sigma Aldrich) was added to obtain a 1:2 molar ratio between Cu/Ti. The mixture was then dried for 1 h at 80 °C. In the next step of the synthesis, both samples were annealed at 730 °C for 10 h (increase rate 5 °C min^–1^) in a 50 mL min^–1^ flow of NH_3_. After slowly cooling to room temperature (5 °C min^–1^), the powdered support was prepared. To deposit Ir nanoparticles, a water solution of IrBr_3_·H_2_O (Alfa Aesar), prepared by dissolving approx. 0.1 mg of IrBr_3_·H_2_O in 1 mL of water at 80 °C, was added to 0.14 mg of the support. The obtained paste was first dried at 50 °C in air and then thermally treated at 120 °C in a 5% H_2_/Ar atmosphere. After 1 h, the temperature was increased to 450 °C (2 °C min^–1^) for an additional 1 h and then decreased to room temperature (3 °C min^–1^) to obtain both samples, Ir/TiON*_x_*/C and Ir/CuTiON*_x_*/C. Weight percentages of compounds obtained by ICP-OES analysis are 11.8% of Ir and 7.4% of Ti in Ir/TiON*_x_*/C; and 14.1% of Ir, 11.5% of Ti, and 6.7% of Cu in Ir/CuTiON*_x_*/C. We note here that the ratio of Ir to Ti is slightly higher in the case of Ir/TiON*_x_*/C (1.58) than that for Ir/CuTiON*_x_*/C (1.2). The increased performance of the latter is thus not an effect of higher Ir loading but better Ir utilization.

### Characterization of Materials

For both samples, elemental analysis was carried out using an inductively coupled plasma-optical emission spectrometry (ICP-OES) instrument (Varian 715-ES). Samples were prepared by a microwave-assisted digestion system (CEM MDS-2000) in 3:1 v/v HCl/HNO_3_ and subsequently diluted with a 2% v/v HNO_3_. Certified, ICP-grade, single-element standards (Merck CertiPUR), diluted with ultrapure water (Milli-Q, Millipore), HNO_3_, and HCl (Merck-Suprapur) were also prepared for the analysis.

XRD spectra were recorded using a Siemens D5000 diffractometer. Diffractograms of the samples were acquired with Cu-K_α1_ radiation with a wavelength of 1.5406 Å in the alpha1 configuration using a Johansson monochromator on the primary side in the 2θ range from 10 to 60°. To identify the phases, the X’Pert HighScore Plus program and the International Centre for Diffraction Data (ICDD) PDF-4+ 2019 database^33^ were used.

XPS measurements were performed using a PHI Quantera II scanning X-ray microprobe with a monochromatic Al Kα X-ray source (1486.6 eV, 15 kV). XPS spectra were recorded on pristine and electrochemically tested catalyst spots: a pass energy of 280 eV and step sizes of 1 eV were employed for survey spectra acquisition, whereas for high-resolution spectra these were 140 and 0.250 eV. The adventitious C 1s peak set to 284.6 eV was employed to energy-correct all high-resolution spectra, processed, and deconvoluted using CasaXPS (version 2.3.22PR1.0). For high-resolution spectra deconvolution, Shirley-type backgrounds and modified functional Lorentzian or Gaussian–Lorentzian line shapes were employed, as reported by Freakley et al. for Ir,^34^ Ti, and Cu.^35^ The specific line shapes employed for the Ir 4f_7/2:5/2_ spin–orbit doublets were LF(0.6,1,150,300) for Ir^0^ and LF(0.5,1.5,25,250) for IrO_2_ × nH_2_O and their related satellites. For Ti 2p_3/2:1/2_ and Cu 2p_3/2:1/2_, the line shapes employed were GL(67) and GL(30), respectively. During fitting, a 4:3 area ratio constraint and 3 eV separation were applied for Ir 4f_7/2:5/2_, whereas, for Ti 2p_3/2:1/2_, the area ratio was constrained to 2:1 and the peak-to-peak separation to 5.6 eV.

Scanning transmission electron microscopy (STEM) analysis was conducted on a Cs-corrected CF-ARM Jeol 200 microscope operated at 80 kV and imaged with a beam current of ∼14.5 pA. Energy dispersion X-ray spectroscopy (EDS) analysis was performed using an SSD Jeol EDS spectrometer. To observe the changes in the nanoparticles after the electrochemical treatment, an identical location transmission electron microscopy (IL-TEM) technique was used. For these experiments, modified floating electrode (MFE) apparatus^36^ was employed. This novel approach allows performing the electrochemical experiment on a TEM grid, which serves as a working electrode. The floating compartment consists of two-piece Teflon housing, which is assembled with Tekka Peek screws. Between these elements, a TEM grid working electrode (Agar Scientific, Holey Carbon Films on 300 Mesh Gold), a gas diffusion layer (GDL, 280 μm thickness) with 40% Teflon weight wet proofing (Toray Carbon paper 090, Fuel CellStore), and two metallic cones with a spring between them are placed on top of each other. A GDL with hydrophobic properties serves as a separator between the electrolyte and metallic cones and spring, which are used as electric contacts of the working electrode. The suspension preparation and electrochemical activation protocol are described in the following section. Samples were prepared by drop-casting 5 μl of the suspension on an Au side of the grid. For IL-TEM experiments, a two-compartment Teflon cell was used. In the first compartment, floating and reversible hydrogen reference (HydroFlex, Gaskatel) electrodes and in the second Pt mesh (GoodFellow 50 mm × 50 mm) counter electrodes were placed. Compartments were separated with a Nafion membrane (Nafion 117, FuelCellStore).

### Electrochemistry

Suspensions of Ir/TiON*_x_*/C and Ir/CuTiON*_x_*/C were prepared with ultrapure water (MilliQ IQ 7000 Merck) and 2-propanol in a ratio 7:1. To prevent detachment, Nafion (Sigma Aldrich, 5 wt %) was added to the suspension, so that the amount of Nafion was 25 wt % of the solid content in the suspension. Before drop-casting 0.2 μl of the suspension onto a glassy carbon plate, the suspension was sonicated for 15 min in intervals (4 s pulse, 2 s pause) on ice to prevent heating. Prior to electrochemical measurement, spots were located with a vertical camera, placed above the scanning flow cell (SFC).^37^ For all measurements, an SFC connected to the ICP-MS was used.^38−40^ A carbon rod and an Ag/AgCl electrode (Metrohm) were used as counter and reference electrodes, respectively. Freshly prepared 0.1 M HClO_4_ (70% Suprapur HClO_4_, Merck), saturated with Ar was used as an electrolyte and purged through the setup with a flow rate of 200 μl min^–1^. The ICP-MS (Perkin Elmer NexION 300× ICP-MS) instrument was calibrated with known amounts of analytes and internal standards (Re^187^, Sc^45^, and Ge^74^). The electrochemical experiment consisted of activation protocol, stability, and activity measurement. Iridium nanoparticles were activated with 100 cycles in the potential range 0.05–1.45 V with a scan rate of 300 mV/s. Stability was evaluated by calculating the S-number, which is defined as the ratio between the amount of total evolved oxygen and dissolved iridium.^41^ The number of oxygen molecules was calculated from the charge at the end of 5 min galvanostatic hold at 5 mA cm^–2^ when the steady dissolution was reached. The activity was measured afterward with a linear scan of potential with 20 mV/s from 1.2 V to the cut-off at 5 mA cm^–2^.

## Results and Discussion

**Figure 1:**
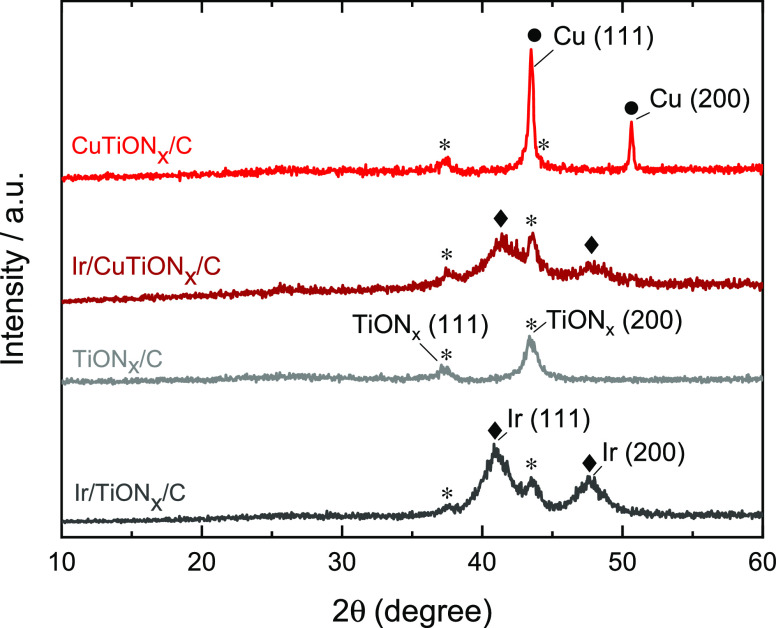
XRD spectra of TiON*_x_*/C (gray), Ir/TiON*_x_*/C (dark gray), CuTiON*_x_*/C (red), and Ir/CuTiON*_x_*/C (dark red).

After each step of the synthesis, the obtained sample was characterized by XRD analysis (Figure 1). Peaks at 37.1° (111) and 43.1° (200) correspond to cubic TiON*_x_* (*, PDF 01-084-4872) and confirm a successful reduction of TiO_2_ in the first steps of the synthesis. This is also in line with the absence of characteristic peaks for TiO_2_ (PDF 01-073-8760). Sharp peaks at 43.4 and 50.6° in the spectra of CuTiON*_x_*/C are assigned to the cubic copper (•, PDF 04-004-6299). After iridium nanoparticles deposition, the two Cu diffractions are not clearly visible anymore, which suggests that the copper’s crystal structure has disappeared. Two new peaks at 40.7° (111) and 47.3° (200) are seen in the spectra of Ir/TiON*_x_*/C and Ir/CuTiON*_x_*/C after the final step of the synthesis, which are attributed to the crystalline cubic iridium (⧫, PDF 04-007-8342). Iridium peaks are broad in both spectra and thus indicate the presence of nanocrystallites in a range of few nanometers.

**Figure 2:**
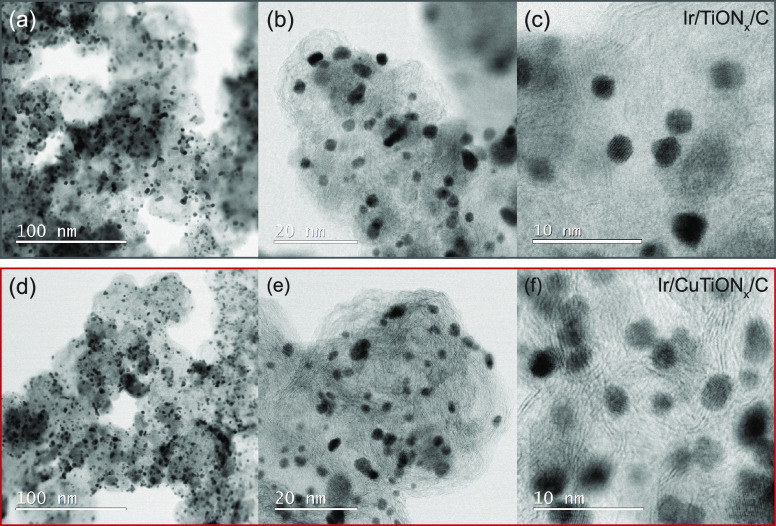
Bright-Field STEM micrographs of Ir/TiON*_x_*/C (top a–c) and Ir/CuTiON*_x_*/C (bottom d–f).

The nanoscale morphological characteristics of both samples were investigated by STEM analysis. The resulting micrographs of Ir/TiON*_x_*/C and Ir/CuTiON*_x_*/C are presented in Figure 2. Similarities between the samples are clearly observable. In both, the presence of finely dispersed small iridium nanoparticles can be confirmed. Particles in the size range of 3–4 nm were mostly detected in Ir/TiON*_x_*/C (Figure S1), while smaller nanoparticles in the size range of 2–3 nm were found in the sample with additional copper in the support (Figure S2).

**Figure 3:**
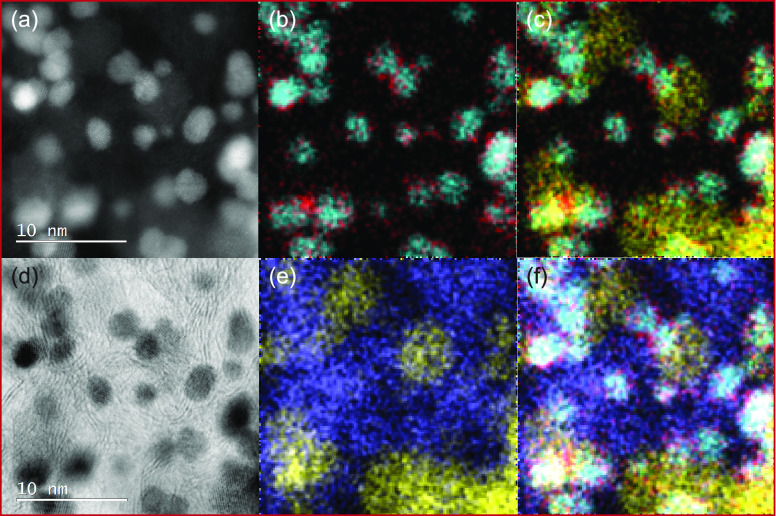
(a) and (d) Annular dark- and bright-field STEM imaging of Ir/CuTiON*_x_*/C nanoparticles, respectively; (b, c, e, f) EDS mapping of Ir/CuTiON*_x_*/C: overlapping signals of Ir (blue), Cu (red), Ti (yellow), and C (purple).

Figure 3 shows the STEM (Figure 3a is dark field and Figure 3d is bright field) and the EDS chemical mapping (Figure 3b,c,e,f) results of the Ir/CuTiON*_x_*/C sample. Interestingly, by overlaying the iridium (blue) and copper (red) signals (Figure 3b), we can see that copper is deposited on iridium nanoparticles in a core–shell-like structure (presented in more detail in Figures S3 and S4). We presume that when iridium ions were added to the CuTiON*_x_*/C support in the second step of the synthesis, a galvanic displacement reaction occurred.^42^ Iridium, having a higher reduction potential than copper, has triggered the spontaneous dissolution of metallic copper, already present in the support (Figure S5). As the paste of the powdered sample and iridium bromide solution was not washed prior to the annealing process, copper ions, dissolved by a reaction of galvanic displacement, were left in the sample and deposited onto metallic iridium particles during the annealing step in reducing atmosphere.

This tentative mechanism could explain the presence of copper, dispersed on the surface of iridium nanoparticles. In Figure 3e,f, the results of EDS mapping of carbon and titanium are presented. Iridium nanoparticles are predominantly dispersed over the TiON*_x_* support, which is shown in more detail in Figures S6 and S7. Distribution over TiON*_x_* is essential for the SMSI effect between Ir and TiON*_x_* to be effective. The addition of carbon into the support enabled a more efficient transformation of TiO_2_ to TiON*_x_* by acting as a reducing agent. Using Ketjen Black with a high surface area (approx. 1300 m^2^/g - BET) was also beneficial for the preparation and fine dispersion of the high-surface-area TiON*_x_*. The possible effect of carbon on the electrocatalytic performance was not part of this study; however, it was recently addressed in a study published by Moriau et al.^43^ The authors have shown that the coverage of reduced graphene oxide nanoribbons with TiON*_x_* has a beneficial effect on the electrocatalytic properties of Ir nanoparticles compared to Ir nanoparticles supported only on reduced graphene oxide nanoribbons.

**Figure 4:**
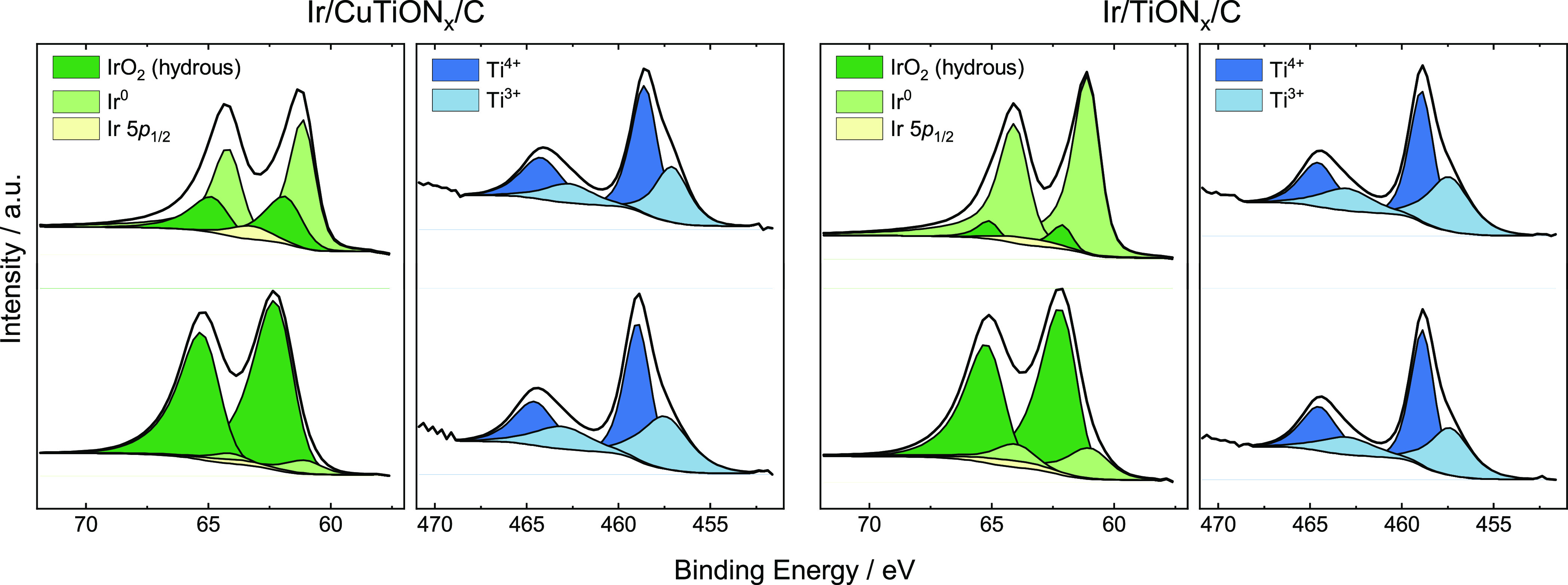
(a) Ir 4f of Ir/CuTiON*_x_*/C and (c) Ir/TiON*_x_*/C, (b) Ti 2p of Ir/CuTiON*_x_*/C, and (d) Ir/TiON*_x_*/C XPS spectra before (top) and after (bottom) the electrochemical experiment.

The oxidation states of the catalysts’ surface species before and after electrochemical experiments were determined by XPS. Ir 4f, Ti 2p, and Cu 2p spectra of Ir/CuTiON*_x_*/C and Ir/TiON*_x_*/C are plotted in Figures 4 and S8. Clearly, Ir is predominantly in a metallic state in the pristine samples (peak at 61 eV) with the partial presence of Ir^4+^ in both Ir/CuTiON*_x_*/C (30%) and Ir/TiON*_x_*/C (8%). The higher concentration of the surface oxide in the sample with Cu seems slightly counterintuitive, as one would expect that the Cu-coverage of Ir particles would suppress the surface oxidation in air; however, it could be related to the different particle sizes of nanoparticles in both samples. After the electrochemical experiment (details below), the Ir surface of both samples is almost completely oxidized, which can be seen from the predominant presence of a peak in the spectra at 62.01 eV. The Cu 2p spectrum (Figure S8) indicates the presence of native oxide on top of a pure metal Cu surface with a relative Cu composition of 44.5% of Cu^0^ and 55.5% of Cu^2+^.^35^ The concentration of Cu on the surface after the experiment was negligible, and thus, the deconvolution of the Cu 2p spectra was excluded from the analysis (as the majority of Cu gets dissolved). Interestingly, the Ti 2p spectrum does not change notably after the experiment. In both samples, Ti is present in Ti^4+^ (458.9 eV) and Ti^3+^ (457.4 eV) forms, which is attributed to TiO_2_, formed on the surface of TiON*_x_*, as it is known to oxidize on air.^44^

**Figure 5:**
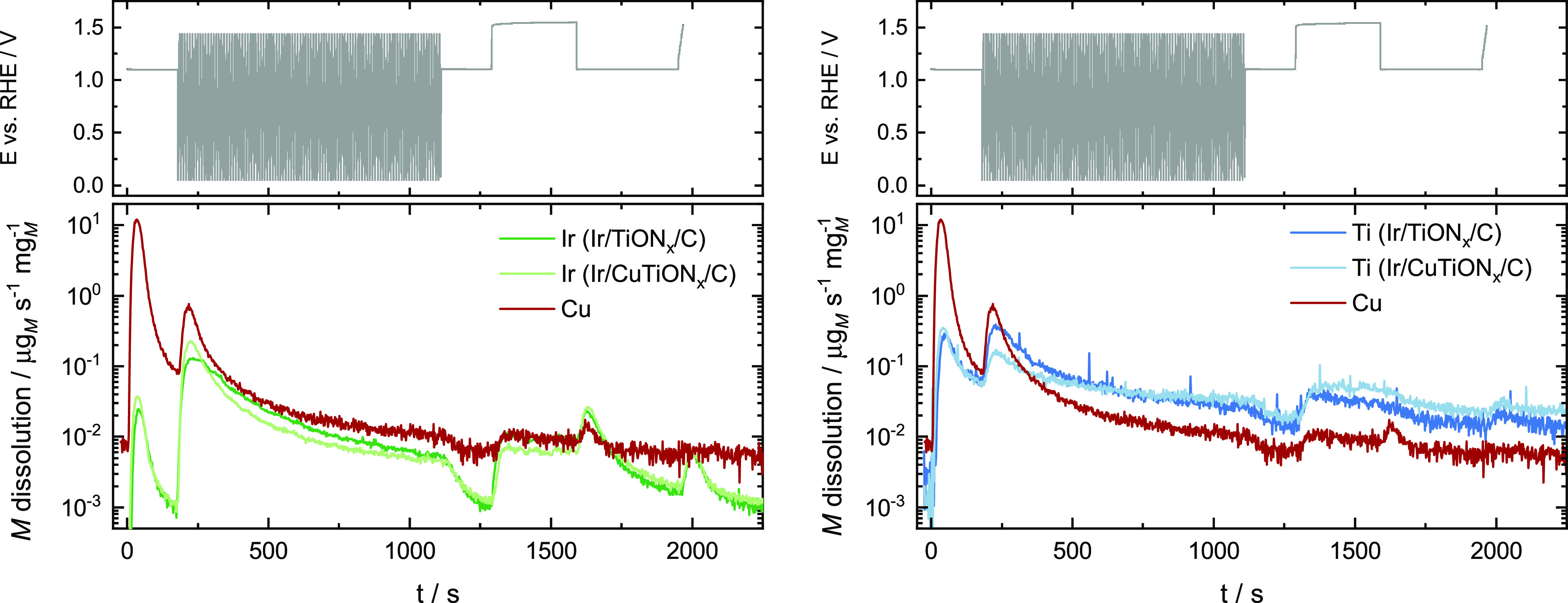
Electrochemical protocol and simultaneous dissolution of iridium, titanium, and copper.

**Figure 6:**
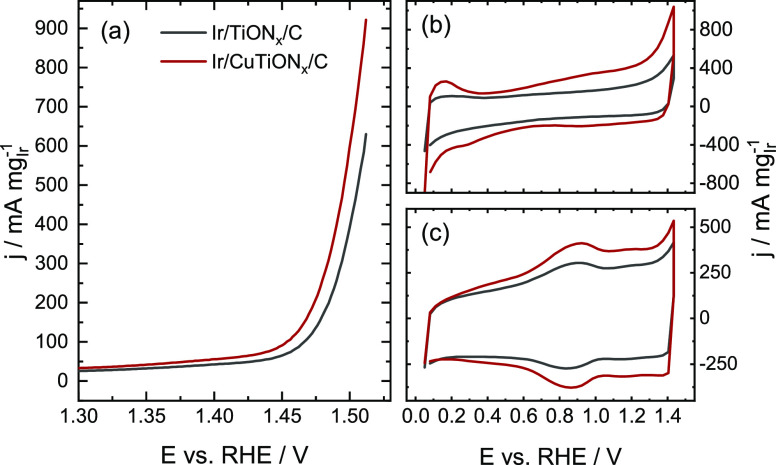
(a) Mass-normalized activity of Ir/TiON*_x_*/C and Ir/CuTiON*_x_*/C, and cyclic voltammograms of electrochemical activation protocol: (b) 1st and (c) 100th cycles.

Electrochemical characterization was performed using an SFC coupled to ICP-MS, which allowed the online detection of dissolved species in the electrolyte. Dissolution of iridium, titanium, and copper is presented together with the electrochemical protocol in Figures 5 and S9. Signals for iridium and titanium in the mass spectra increased immediately after the contact with the electrolyte. This was due to the chemical dissolution of surface defects and the passivation of TiON*_x_*.^45,46^ Upon contact of Ir/CuTiON*_x_*/C with the electrolyte, the intense dissolution of copper was detected as well as expected due to its instability at a relatively high initial potential and the possible presence of oxygen in the electrolyte. With fast cycling in a broad potential window (0.05–1.45 V) during activation, increased dissolution of both iridium and titanium was observed. It is attributed to their transient dissolution, occurring when metals are exposed to oxidation and reduction.^25,47^ The dissolution rate decreases significantly with time for both iridium and titanium due to the development of a protective stable passive oxide layer on their surfaces.^6,48,49^ This hypothesis is confirmed by the Ir 4f XPS spectra of both samples (Figure 4) and cyclic voltammograms of the first and the last cycle of activation plotted in Figure 6b,c. In both CVs, the *H*_upd_ characteristic peak between 0.05 and 0.3 V in the first cycle indicates the presence of a metallic iridium surface at the beginning of the electrochemical experiment.^48,50^ The metallic state was expected, as no oxidation step was introduced in the synthesis procedure. After the activation protocol, the *H*_upd_ feature disappeared in the CVs of both samples. Instead, two broad peaks around 0.9 V_RHE_ in the anodic and cathodic branches of the CV appeared. They can be attributed to the Ir(III)/Ir(IV) transition, typically observed in CVs of hydrous or amorphous iridium oxides.^13,48,51,52^ Iridium surface oxidation is directly related to the dissolution behavior, observed during fast cycling.

Interestingly, dissolution was also detected for titanium, which proves that titanium oxynitride undergoes transient dissolution as well. Judging by other known examples of transient dissolution,^47,53^ it is reasonable to conclude that the titanium oxynitride is passivated by the oxide layer made by both the exposure to air and electrochemical activation. A slightly higher dissolution of iridium during activation was detected in the Ir/CuTiON*_x_*/C sample. This could be due to concomitant copper dissolution. As copper is dispersed on iridium nanoparticles and parts of it are in contact with TiON*_x_* as well, dissolution of approximately 65% of the initial amount of copper (Table S1) might have triggered additional iridium dissolution or even detachment. However, a closer look into the CVs in Figure 6 offers another explanation. The observed *H*_upd_ peak in the first cycle is more resolved in the Ir/CuTiON*_x_*/C analogue. The difference in both CVs and dissolution profiles could thus also originate from the different surface areas for the two iridium-based catalysts studied. The noted difference in *H*_upd_ peaks between samples indicates a higher surface area of Ir in the copper-containing material. The consequently higher number of iridium atoms exposed to oxidation and reduction in the case of Ir/CuTiON*_x_*/C can explain the observed enhanced dissolution. Mass-normalized CVs of both samples after activation show a higher Ir-based surface area of the sample with copper, namely better Ir utilization. This is in line with smaller Ir nanoparticles.

The higher surface area can also be a result of the formation of more “porous” or exposed Ir nanoparticle structures after leaching of copper at contact with the acidic electrolyte and electrochemical activation.

**Figure 7:**
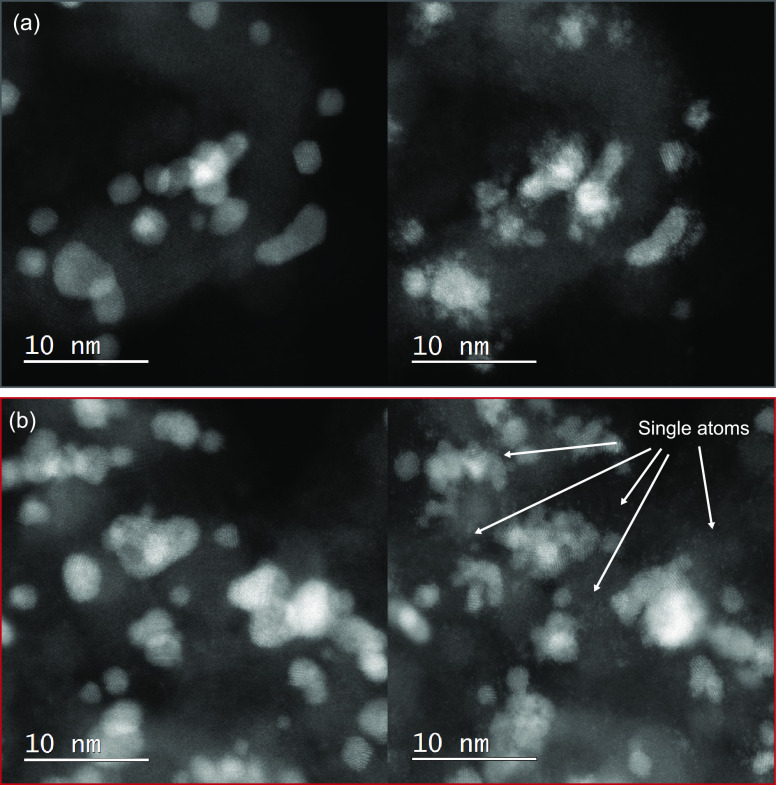
IL-STEM ADF images of (a) Ir/TiON*_x_*/C and (b) Ir/CuTiON*_x_*/C before (left) and after (right) activation with 100 cycles, 300 mV/s, and in the potential range 0.05–1.45 V.

**Figure 8:**
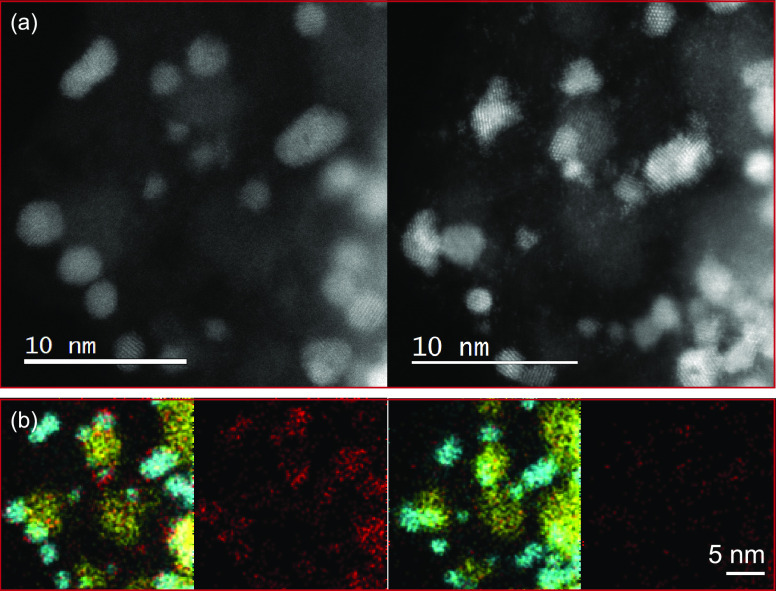
IL-STEM ADF images of Ir/CuTiON*_x_*/C (a) before (left) and after (right) activation with the formation of single atoms (left) and (b) EDS chemical mapping of Ir (blue), Cu (red), and Ti (yellow).

To prove this hypothesis, IL-TEM experiments were conducted before and after the electrochemical activation protocol (0.05–1.45 V, 300 mV/s, 100 cycles). The obtained images of Ir/TiON*_x_*/C and Ir/CuTiON*_x_*/C are presented in Figure 7. It can be seen that fast cycling resulted in visible structural changes of Ir nanoparticles in both samples. As expected, based on CVs, the increased surface area of Ir can be confirmed. The formation of an amorphous layer on the predominantly larger Ir nanoparticles in Ir/TiON*_x_*/C can be seen in Figures 7a and S10. Here, the initial shape of Ir nanoparticles seems to be preserved and only minor movements of individual particles were observed. Similarly, the formation of an amorphous layer was also detected on the images of Ir/CuTiON*_x_*/C, presented in Figures 7b and S11. We wish to note here the possibility that, in both samples, the amorphous oxide layer could be partially reduced due to potential instability under the electron beam used in the TEM investigation (time-dependent modifications of the amorphous layer under an electron beam presented in more detail Figure S12) and we might be therefore unable to resolve its exact structure and extent.^54^ Despite similarities between both samples, it can be suggested that the dissolution of Cu that accompanied electrochemical activation of Ir/CuTiON*_x_*/C triggered a more pronounced fragmentation of Ir nanoparticles that were initially covered with Cu. It can be also assumed that leaching of Cu from the surface of Ir particles initiated the formation and adsorption of Ir single atoms on the support, which can be clearly seen in Figure 8a. Interestingly, notably, fewer single atoms were observed in the case of Ir/TiON*_x_*/C (see Figures 7, S10, and S13). While both the higher degree of fragmentation and formation of single atoms contribute to the higher electrocatalytically active surface area of Ir/CuTiON*_x_*/C, it was nevertheless concluded that this phenomenon is predominantly a result of smaller nanoparticles in Ir/CuTiON*_x_*/C. With this conclusion, the beneficial effect of Cu on the utilization of Ir can be confirmed. Additionally, based on the results of chemical mapping, presented in Figure 8b, it can be assumed that most of Cu were dissolved during the electrochemical treatment and that after the experiment its residues are homogeneously dispersed through the support and to a smaller extent concentrated in the cores of Ir nanoparticles. This could explain the slightly higher dissolution of Ti in the Ir/CuTiON*_x_*/C sample during the galvanostatic hold experiment.

Activities of both samples were measured in the last step of the experiment with an LSV from 1.2 V to a cutoff geometric current density of 5 mA/cm^2^. Mass activities are presented in Figure 6a and Table 1. Ir/CuTiON*_x_*/C displays approximately 35% higher activity than Ir/TiON*_x_*/C. The difference can be attributed to the higher amount of the OER active sites in the case of Ir/CuTiON*_x_*/C, as the addition of Cu to the sample resulted in the formation of smaller Ir nanoparticles. The enhanced activity could be also an effect of the residual copper left in the iridium oxide lattice. Reier et al. reported a somewhat similar observation for the Ir–Ni system.^16^ They showed that after the leaching of nickel, its value in the mixed oxide leveled at approximately 12% after OER catalysis, likely due to stabilization through interaction with iridium. The residual nickel in the lattice may contribute to electronic and geometric effects, which are beneficial for the water-splitting catalysis. In our case, the presence of a minor amount of copper in the structure during OER operation is confirmed both from the IL-TEM and ICP-MS results, where the signal for copper increased after galvanostatic hold simultaneously with the iridium signal even after the harsh activation cycling shown to significantly leach copper. This suggests that iridium has covered the surface of the nanoparticle while copper is still left in the near-surface areas of the sample, i.e., below the Ir-rich surface, after activation. The increased dissolution, following the end of the galvanostatic hold, could be explained by the partial reduction and subsequent transient dissolution of the formed iridium oxide.^55^ In previous reports on Ir–Cu systems, enhanced activity toward the OER was attributed not only to the increased surface area but also to the change in the IrO_2_ structure due to the uniform replacement of Ir^4+^ by larger Cu^2+^ ions in the IrO_2_ crystal structure^18^ and tuning the electron occupation between the t_2g_ and e_g_ orbital states of Ir sites.^56^ We, however, note that our material outperforms all Ir–Cu mixed oxides reported in the literature (Table S2).^18,56,57^ Therefore, it is reasonable to ascribe the enhanced activity, in addition to the effect of copper, also to the SMSI effect.^21,32^ However, this effect should positively affect both samples.

**Table 1:** Mass-Normalized Activities and Tafel Slopes^a^

sample	mass activity at 1.51 V vs RHE [mA mg^–1^]	charge-normalized activity at 1.51 V vs RHE [mA mC^–1^]	Tafel slope [mV dec^–1^]
Ir/TiON*_x_*/C	626 ± 49	10.7 ± 0.2	58.3 ± 0.3
Ir/CuTiON*_x_*/C	840 ± 33	10.7 ± 0.3	58.0 ± 0.9

a Averaged over 3 measurements.

To get more insight into the mechanism of the OER on Ir/TiON*_x_*/C and Ir/CuTiON*_x_*/C, Tafel analysis was performed. Tafel slopes (58 mV dec^–1^, Table 1 and Figure S14) for both samples indicate the same rate-determining step and thus no mechanistic difference in the OER between the samples. The difference in mass activity can be thus not attributed to the intrinsic effects but rather to the higher amount of the OER active sites of the copper analogue. Mass normalization was chosen as it is industrially the most important parameter, which most reliably describes the activity and can be easily compared with the literature data. However, it does not show intrinsic activity. To evaluate the latter, a redox peak between 0.6 and 1.1 V was integrated, as the charge is directly correlated to the number of active sites.^58−60^ It is important to emphasize that the use of capacitive current as a method for ECSA measurement (as commonly done) is not suitable in the case of supported analogues as it does not consider the contribution of the support to it.^61^ To eliminate the contribution of the support, only the peak was integrated, eliminating the capacitive current (Figure S15). Interestingly, charge-normalized activities (Table S1 and Figure S16) are, contrary to mass-normalized, the same for both catalysts, which again confirms that the higher mass activity of the Cu analogue is predominantly a result of higher surface area. It also suggests that conclusions of previous studies on Ir–Cu systems discussed above are not applicable to this report.

**Figure 9:**
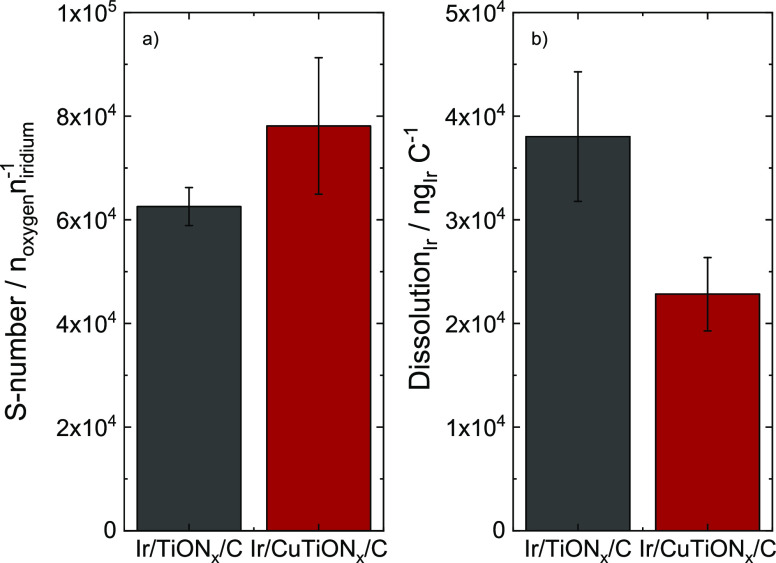
(a) S-numbers and (b) active surface area normalized dissolution of iridium for Ir/TiON*_x_*/C and Ir/CuTiON*_x_*/C (averaged over 3 measurements).

Galvanostatic polarization at 5 mA cm^–2^ for 5 min was performed to evaluate the stability of both samples by S-numbers, recently reported as a stability metric.^41^ The S-numbers (Figure 9a) were estimated from the amount of produced oxygen assuming a 100% faradaic efficiency, divided by the integrated amount of dissolved metal under steady-state conditions. It should be noted that the still ongoing dissolution of Cu negligibly contributes to the overall OER current. Importantly, the S-number enables stability comparison of newly synthesized materials to other OER catalysts as is independent of loading, surface area, and the number of active sites. A comparison of both S-numbers (Figure 9a) shows that the stability of both samples is very similar, with a slightly higher value for the copper analogue. Both numbers are in good agreement with average values, reported for hydrous iridium oxide in the literature.^41,62^ To determine the “intrinsic” stability of the catalysts, the amount of dissolved iridium was normalized to the active surface area reflected by charge (Figure 9b). In this case, the difference between both analogues is significant. Improved stability of Ir/CuTiON*_x_*/C can be explained with the enhanced SMSI effect in this sample. Based on DFT calculations, it was recently shown that the adhesion of single atoms is the strongest and that the magnitude of adhesion energy decreases with increasing the Ir particle size.^30^ Following this, we can conclude that smaller particles and also single atoms present in the Ir/CuTiON*_x_*/C sample are more efficiently stabilized with the SMSI effect than slightly larger particles in Ir/TiON*_x_*/C. This is in line with the study where extremely low Ir loading on the TiON*_x_* film exhibited enhanced OER stability.^30^ We can thus confirm that, despite the initial enhanced dissolution of Ir due to concomitant Cu dissolution, the presence of copper in the structure was beneficial both for activity and stability, as it enabled the formation of smaller nanoparticles with higher surface area, which were also more efficiently stabilized with the SMSI effect.

Dissolution measurements of Ti have shown that the addition of copper does not significantly influence the stability of the support. A tentative explanation for this observation is that copper’s initial dissolution mostly affects iridium, as it is initially concentrated on the noble metal’s nanoparticles. Low amounts of the dissolved titanium additionally confirm that, if ongoing, carbon corrosion does not significantly influence the dissolution of either support or iridium and that despite the difference between both samples, the amount of the dissolved titanium was in the range of few percentages (Table S1). This highlights the stability of the TiON*_x_* support and its applicability as possible support for OER catalysts in the PEM electrolyzer; however, for industrial applications, it would be necessary to develop a synthesis procedure for a high-surface-area TiON*_x_*/C support without carbon, as its instability would influence the long-term corrosion resistivity of the support.

## Conclusions

In this report, we have presented the synthesis and characterization of two analogues of the iridium electrocatalyst supported on titanium oxynitride, dispersed over the high-surface-area Ketjen Black. In the first, iridium nanoparticles with an average size of 3–4 nm were deposited on the TiON*_x_*/C support. To prepare smaller nanoparticles with the enhanced SMSI effect, sacrificial Cu was added to the support prior to iridium deposition in the second analogue, which resulted in the formation of smaller nanoparticles with an average size of 2–3 nm. Based on the XRD, STEM, EDS, XPS, and SFC-ICP-MS results, we have shown that copper had a beneficial effect both on the OER activity and stability of the catalyst. Stability was estimated using S-numbers as stability metrics. The calculated S-numbers of both were in the range of amorphous iridium oxides. After the initial leaching of copper, the oxidized Ir structure stabilized and was found to be approximately twice as durable under OER conditions than the undoped analogue. The mass-normalized activity of the copper analogue was found to be higher than the activity of the undoped catalyst, which was attributed to the higher surface area of iridium. Activities were additionally normalized by a charge the under Ir(III)/Ir(IV) peak to get insight into the intrinsic properties of the catalysts. We have confirmed that higher mass activity for Ir/CuTiON*_x_*/C is predominantly a surface area effect as charge-normalized activities were the same. This is an important breakthrough as it provides a general way to increase the Ir mass activity without sacrificing its stability.

## Supporting Information Available

Particle size distribution; EDX maps and STEM images of Cu/TiON*_x_*/C; Cu 2p XPS spectra; additional electrochemical and ICP-MS analysis; and IL-TEM images of Ir/CuTiON*_x_*/C and Ir/TiON*_x_*/C (PDF)
