# Supplementary material for: Sacrificial Cu Layer Mediated the Formation of an Active and Stable Supported Iridium Oxygen Evolution Reaction Electrocatalyst
Source: ACS Catal. 2021 Sep 28;11(20):12510–9. doi: 10.1021/acscatal.1c02968 (PMC8524421; doi:10.1021/acscatal.1c02968)
Supplement: Supplementary file 1 — cs1c02968_si_001.pdf [file cs1c02968_si_001.pdf]

Supporting information:

Sacrificial Cu Layer Mediated Formation of Active and Stable Supported Iridium Oxygen Evolution Reaction Electrocatalyst

Anja Lončar, [*,a,b] Daniel Escalera-López,[c] Francisco Ruiz-Zepeda,[a] Armin Hrnjić,[a,b] Martin Šala,[d] Primož Jovanovič,[a] Marjan Bele,[a] Serhiy Cherevko,[c] Nejc Hodnik[*,a,b]

[a] Department of Materials Chemistry, National Institute of Chemistry, Hajdrihova 19, 1000 Ljubljana, Slovenia

[b] University of Nova Gorica, Vipavska 13, 5000 Nova Gorica, Slovenia

[c] Helmholtz-Institute Erlangen−Nürnberg for Renewable Energy, Forschungszentrum Jülich, Egerlandstrasse 3, 91058 Erlangen, Germany

[d] Department of Analytical Chemistry, National Institute of Chemistry, Hajdrihova 19, 1000 Ljubljana, Slovenia

* Corresponding Authors: Anja Lončar (anja.loncar@ki.si), Nejc Hodnik (nejc.hodnik@ki.si)

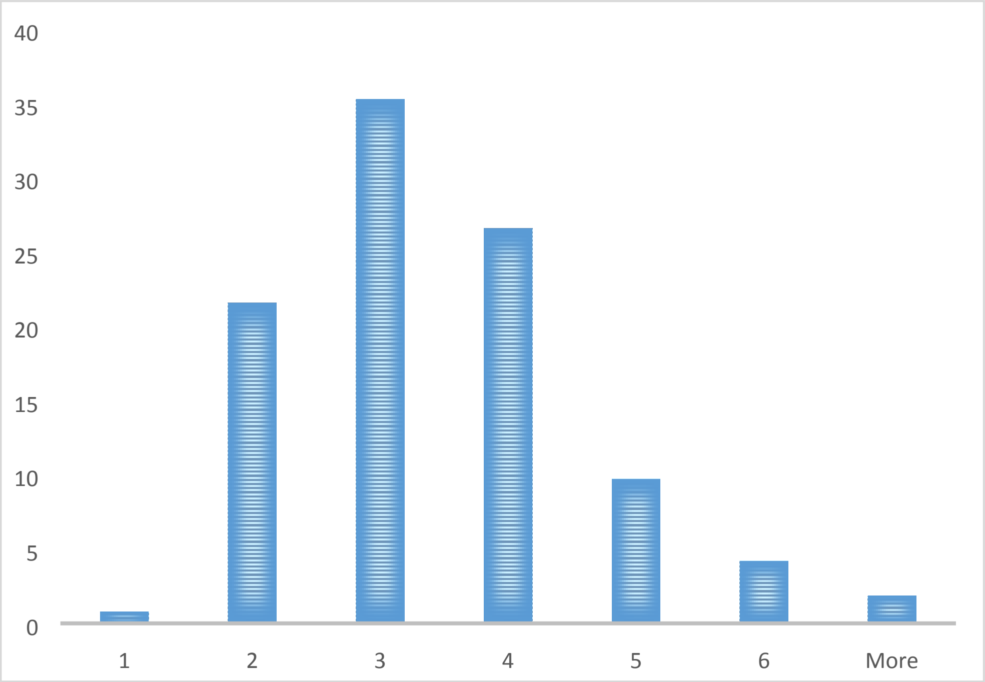

Figure S1: Particle size distribution of $Ir/TiON_x/C$.

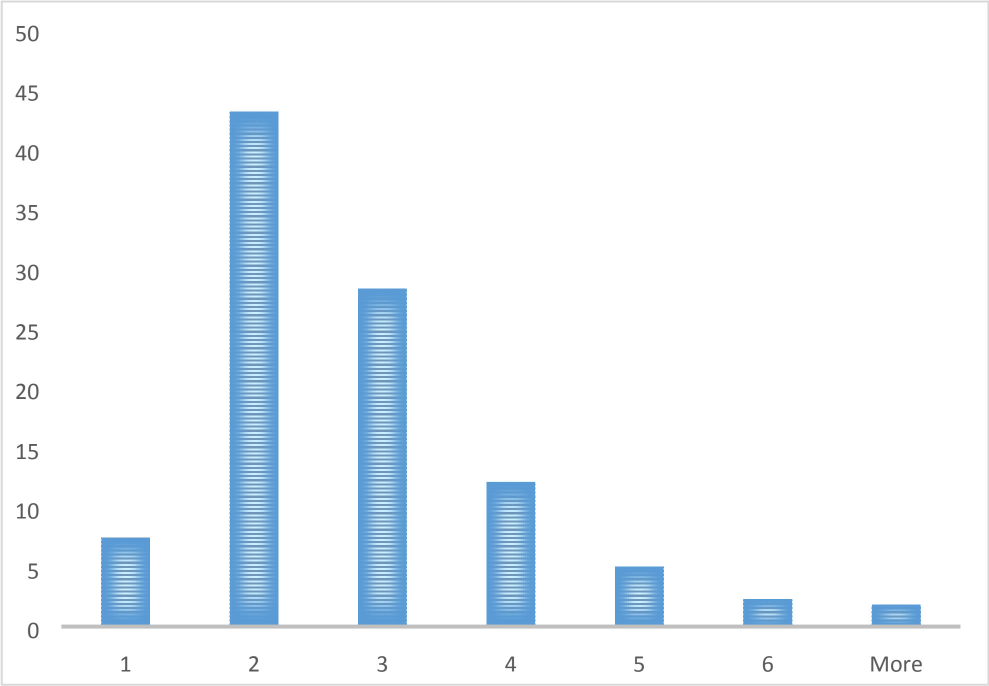

Figure S2: Particle size distribution of Ir/CuTiON$_x$/C.

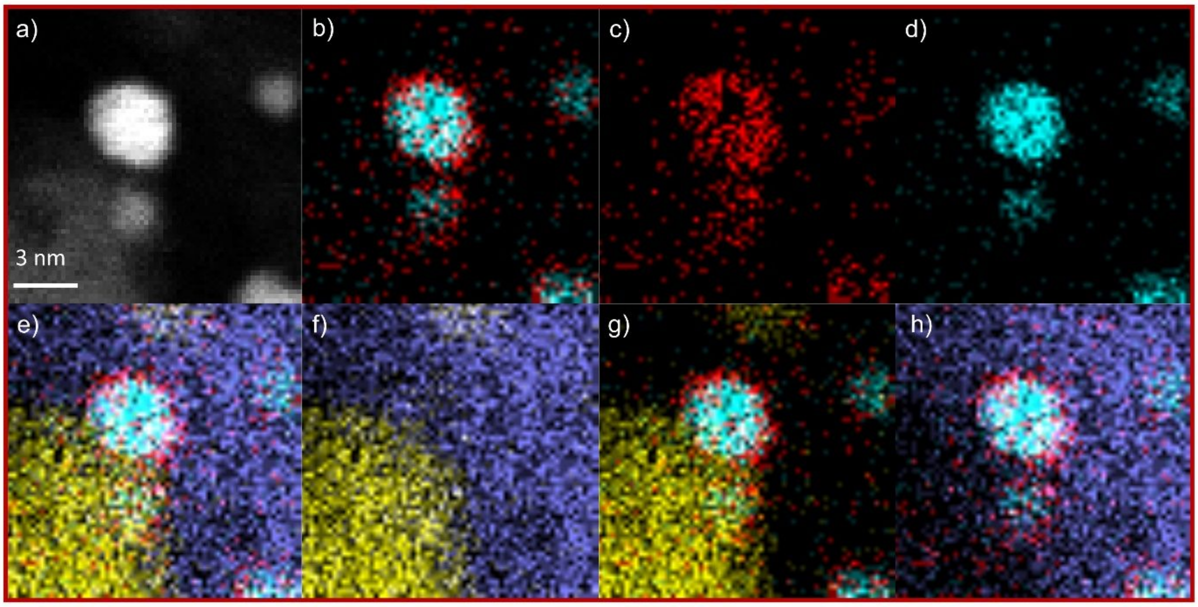

Figure S3: STEM-ADF and EDS mapping of an Ir particle covered by Cu in a core-shell fashion. The particle sits on the TiON$_x$ support.

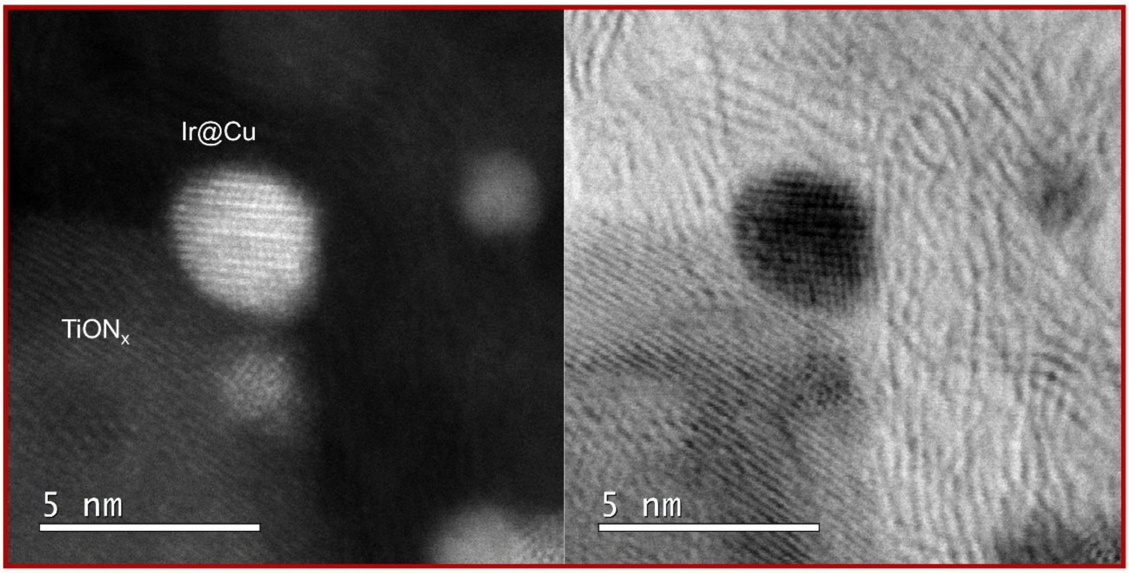

Figure S4. STEM ADF and BF images of an Ir-Cu core-shell particle resting on the $TiON_x$ support.

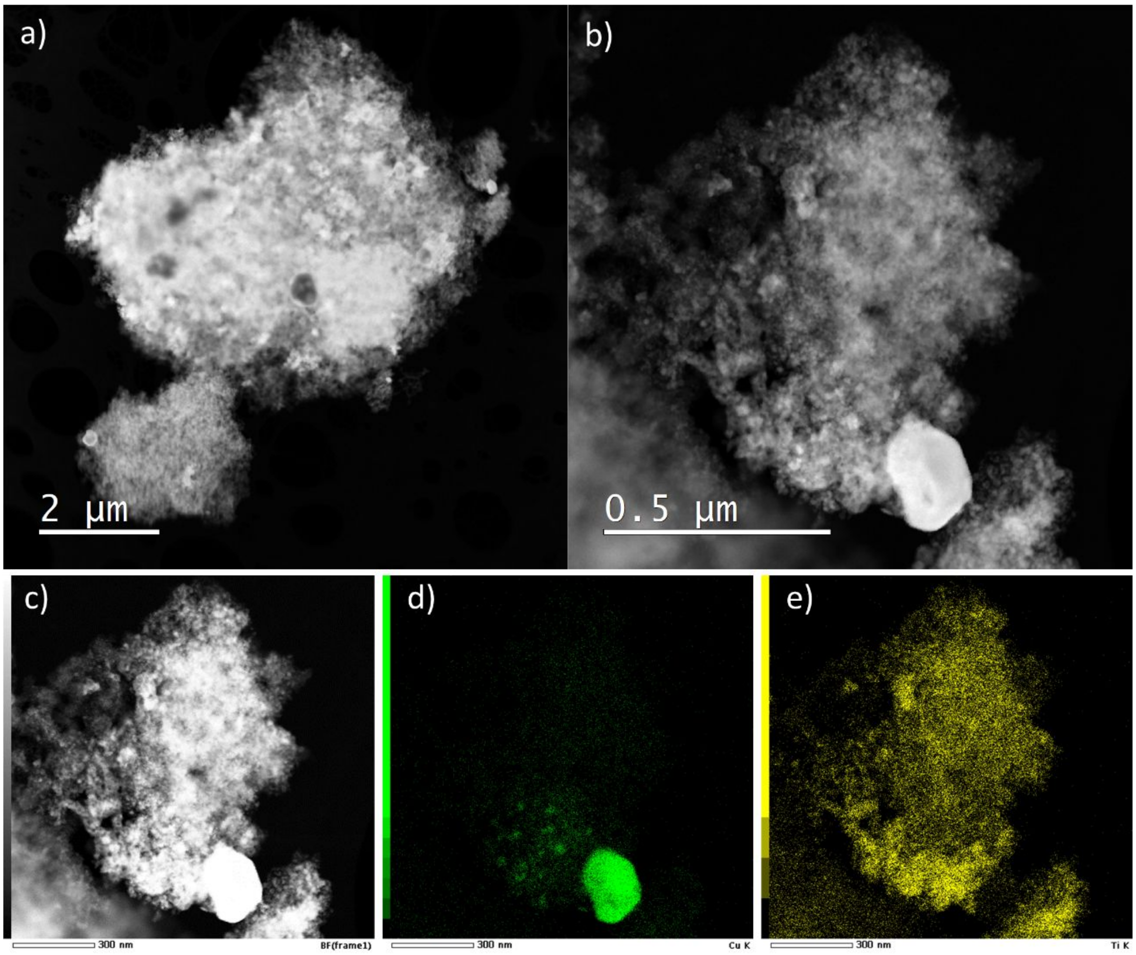

Figure S5: a, b, c) STEM ADF images of support $CuTiON_x/C$ prior to Ir nanoparticles deposition and d, e) EDS mapping of Cu (green) and Ti (yellow).

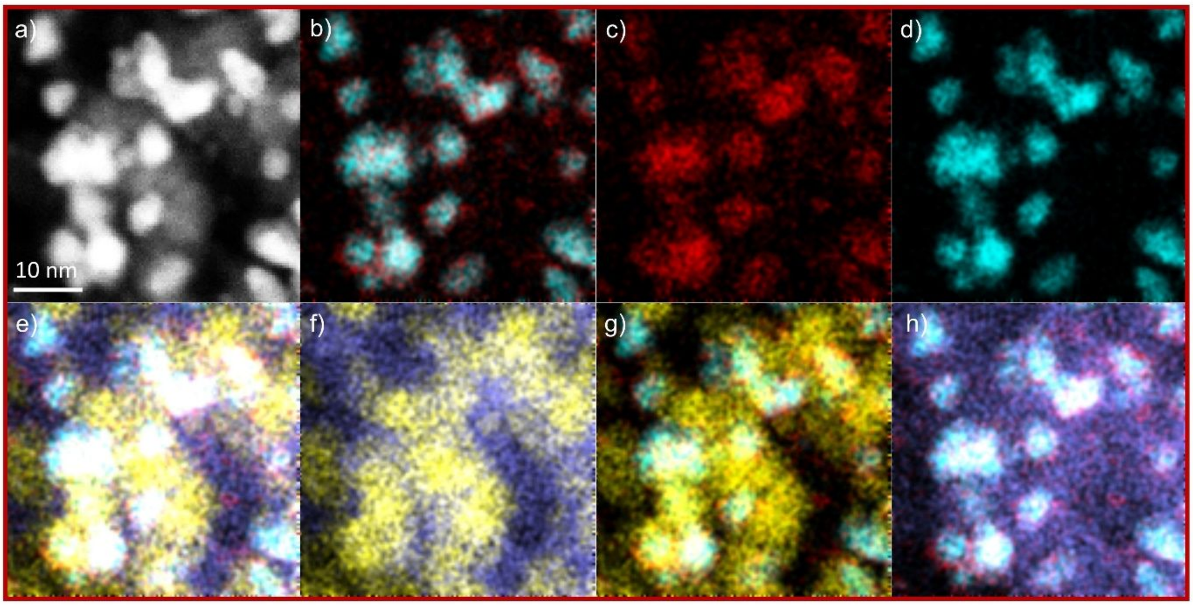

Figure S6. STEM-ADF and EDS mapping of several Ir particles covered by Cu in a core-shell fashion. Most of the Ir@Cu particles rest on the $TiON_x$ support.

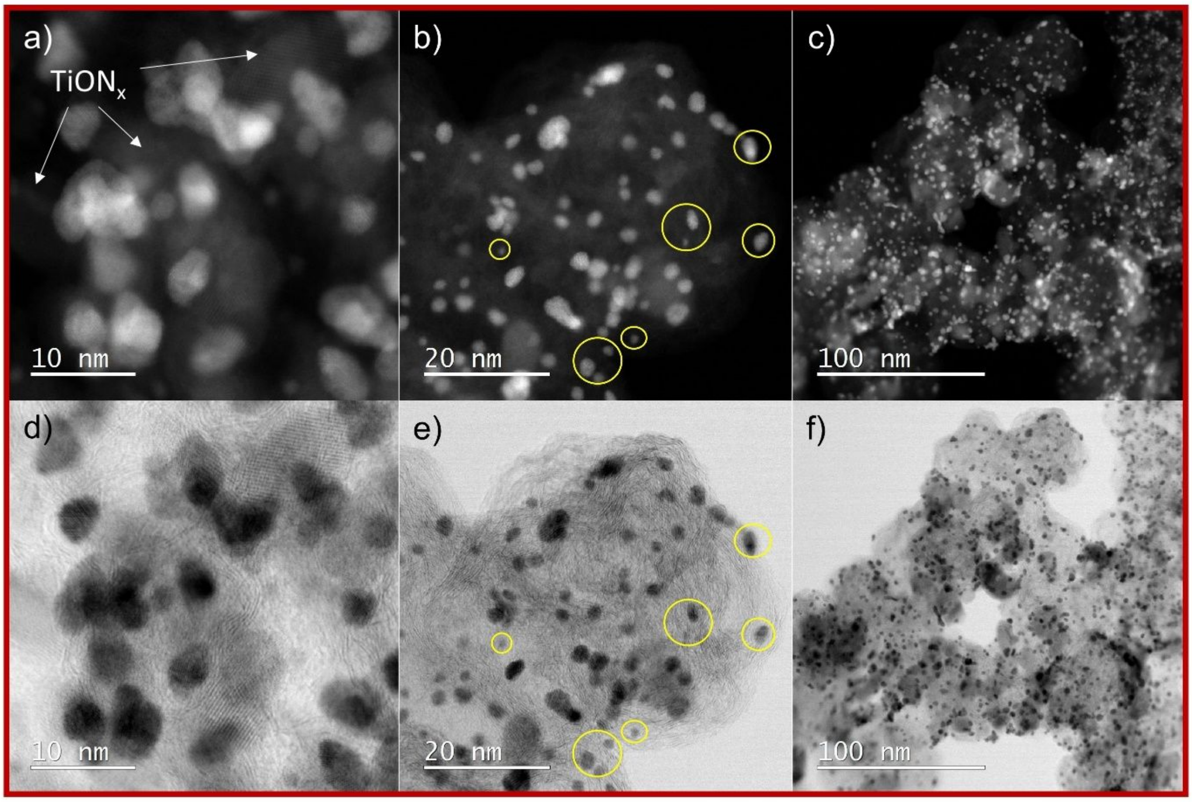

Figure S7. STEM-ADF and BF imaging of the distribution of particles over the support in three different regions and magnifications. ADF and BF images (a, d) correspond to the EDS mapping area from Figure S6. By inspecting both the Z contrast and BF images it is possible to distinguish the $TiON_x$ support, as confirmed by EDS mapping. In the second set of images (b, e), only a small proportion of particles that lay outside of the $TiON_x$ support are identified. A larger field of view is displayed in the third pair of images (c, f) revealing a distribution of particles with a tendency to attach to the $TiON_x$ support.

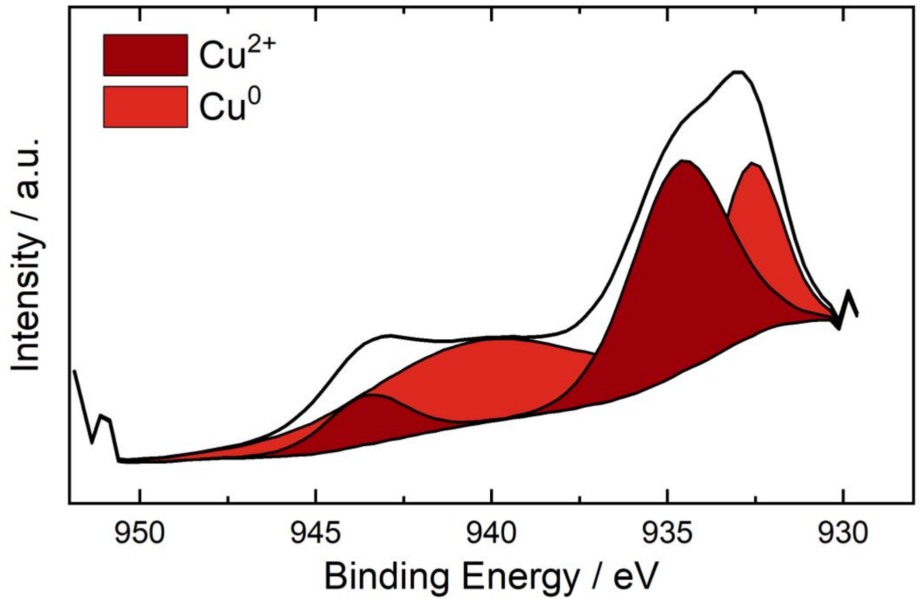

Figure S8: Cu 2p XPS spectra of Ir/CuTiON$_x$/C before the electrochemical experiment.

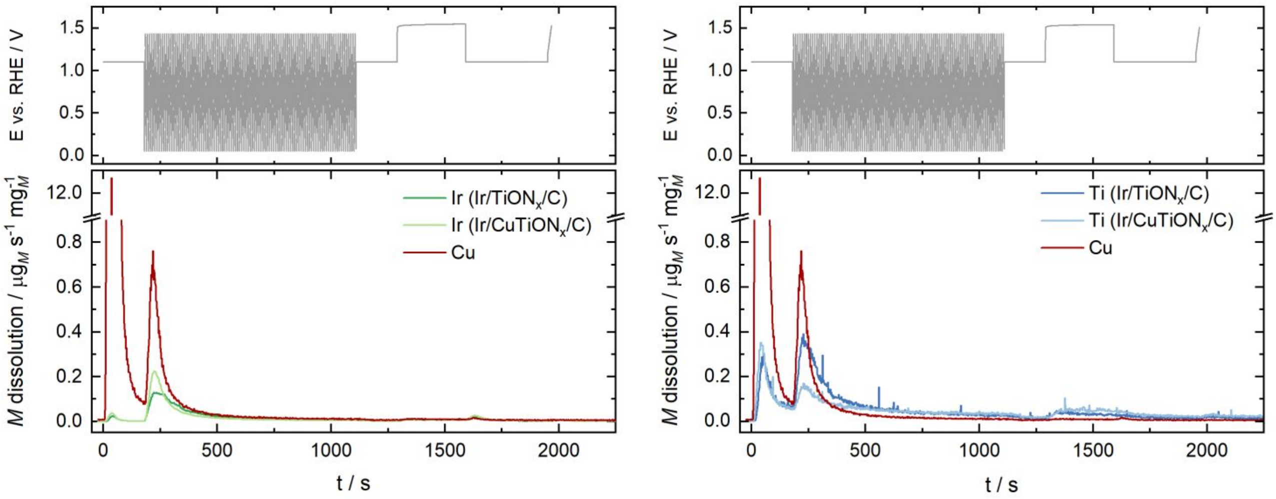

Figure S9: Electrochemical protocol and simultaneous dissolution of iridium, titanium and copper; dissolution plotted on a linear scale.

Table S1: Relative loss of Ir, Ti and Cu after each phase of the electrochemical experiment in Ir/TiON$_x$/C and Ir/CuTiON$_x$/C.

**Metal**	**Sample**	**Contact peak [%]**	**Activation [%]**	**Stability [%]**	**Activity [%]**
**Ir**	Ir/TiON$_x$/C	0.117 ± 0.007	2.7 ± 0.2	0.42 ± 0.04	0.041 ± 0.002
	Ir/CuTiON$_x$/C	0.18 ± 0.03	2.9 ± 0.4	0.43 ± 0.07	0.044 ± 0.008
**Ti**	Ir/TiON$_x$/C	2.2 ± 0.2	6.9 ± 0.7	0.9 ± 0.1	0.19 ± 0.02
	Ir/CuTiON$_x$/C	2.6 ± 0.2	4.3 ± 0.6	1.3 ± 0.1	0.2 ± 0.01
**Cu**	Ir/CuTiON$_x$/C	65 ± 7	9 ± 2	0.29 ± 0.09	0.02 ± 0.01

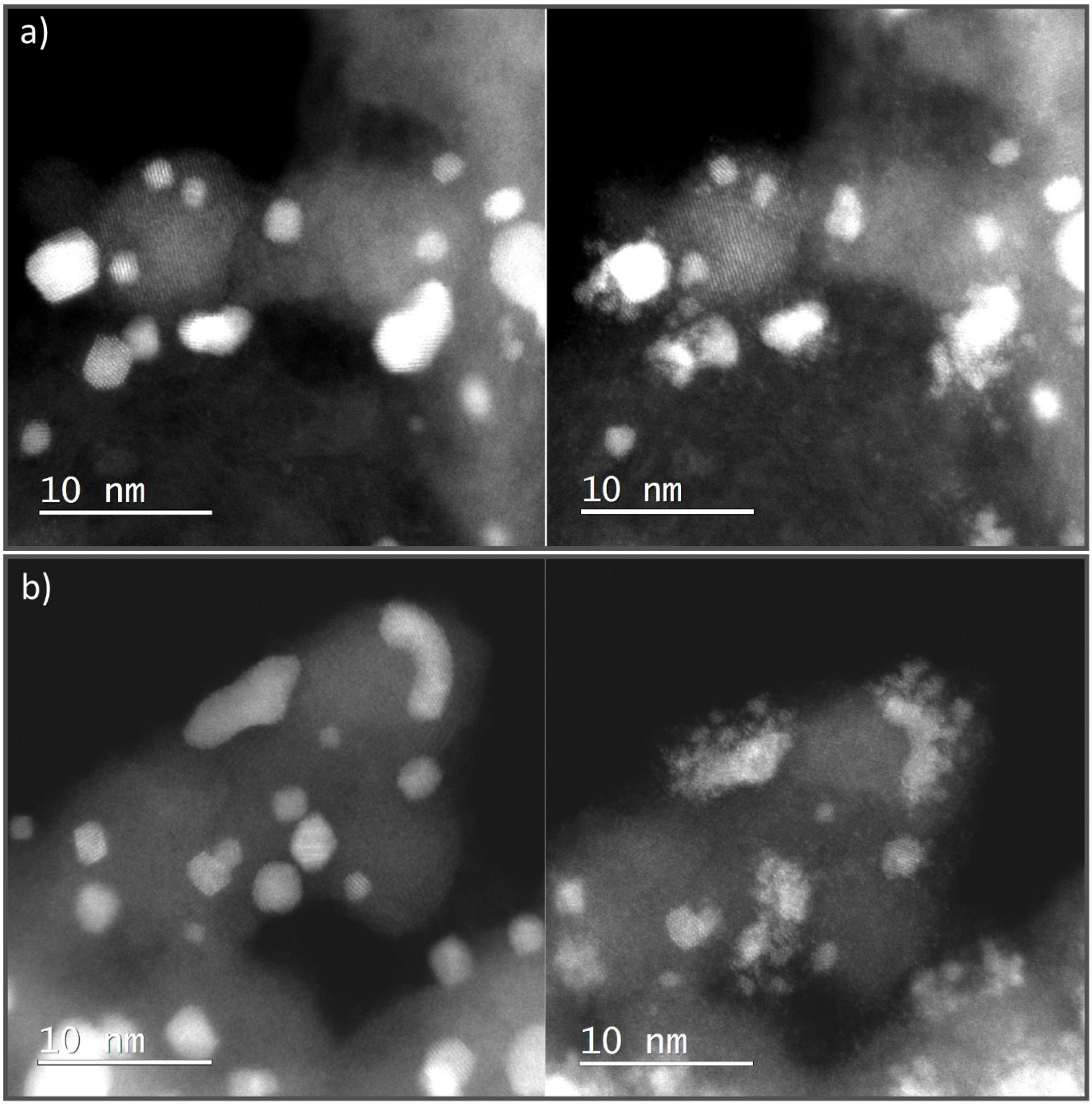

Figure S10: IL-STEM ADF images of two different locations of $Ir/TiON_x/C$; before (left) and after (right) activation with 100 cycles, 300 mV/s in the potential range 0.05-1.45 V.

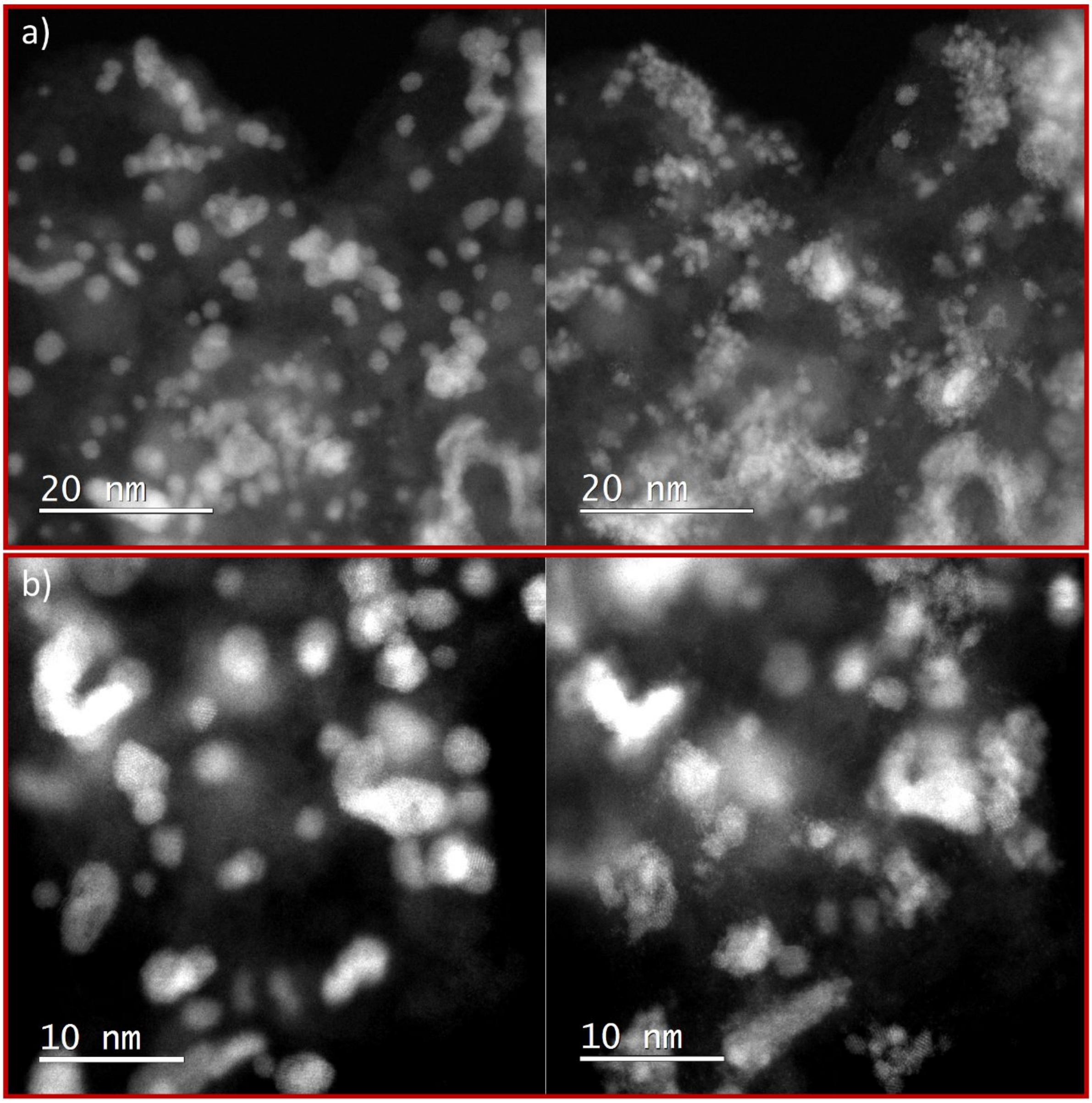

Figure S11: IL-STEM ADF images of two different locations of Ir/CuTiON$_x$/C; before (left) and after (right) activation with 100 cycles, 300 mV/s in the potential range 0.05-1.45 V.

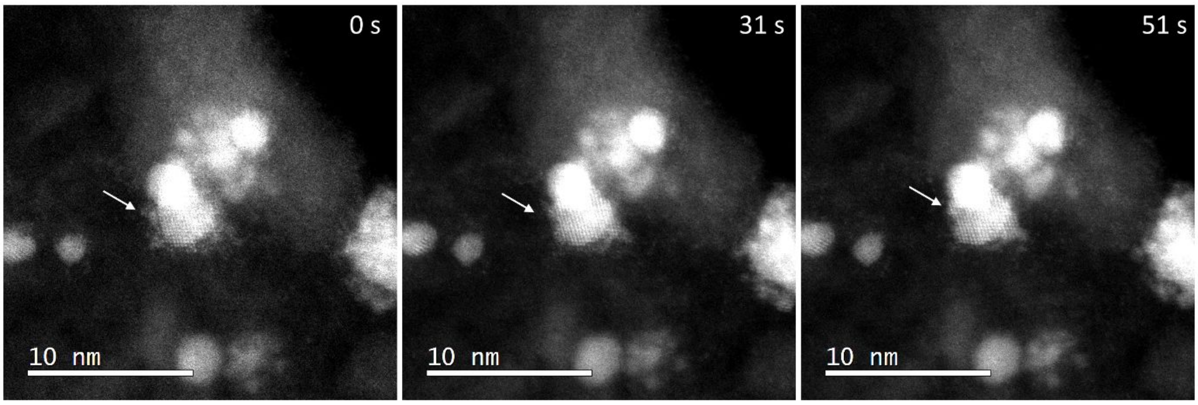

Figure S12: Modifications of the amorphous layer under electron beam (Beam exposure: dose per image 66700 eÅ^2)

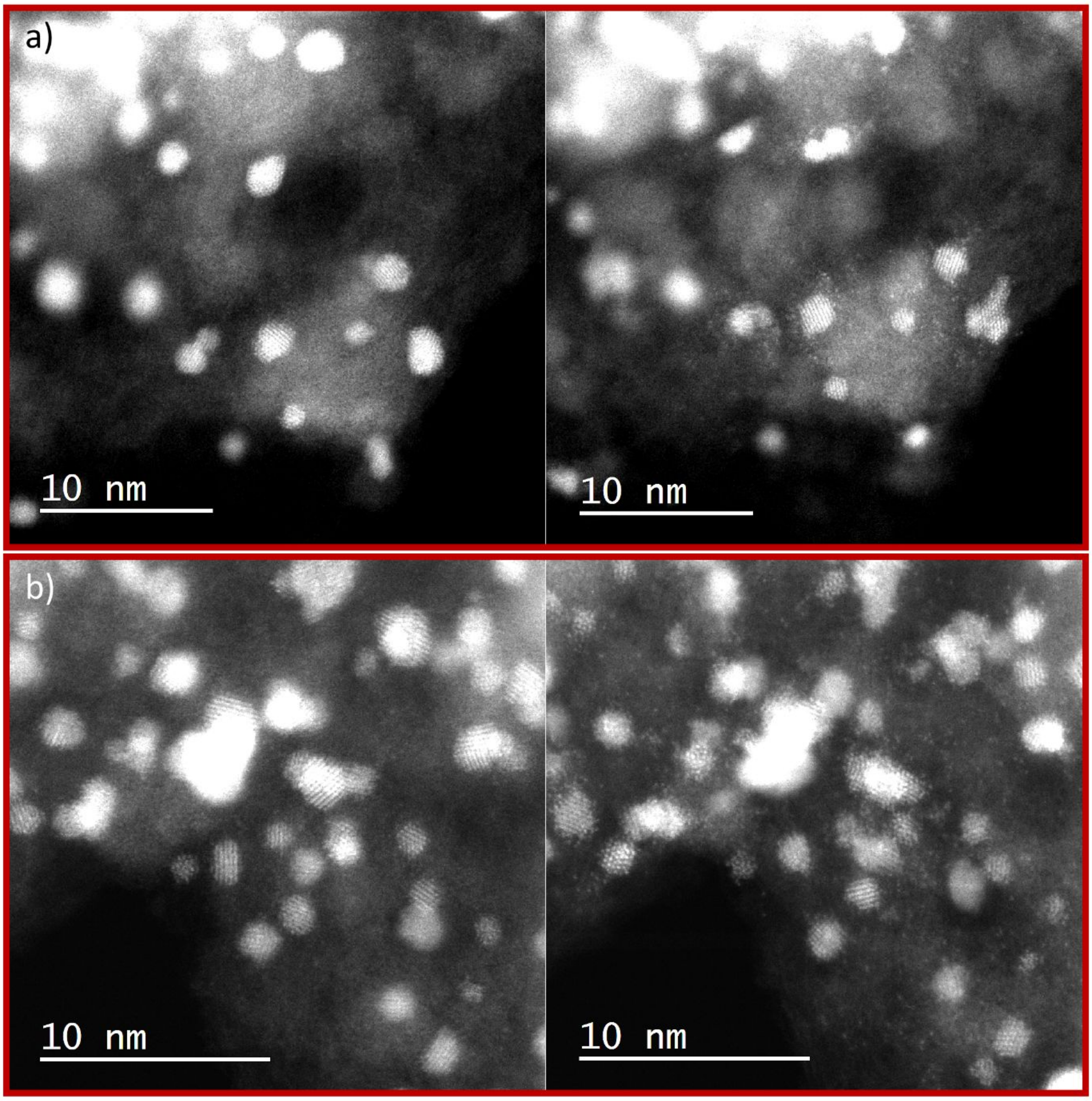

Figure S13: IL-STEM ADF images of Ir/CuTiON$_x$/C before (left) and after (right) activation with the formation of single atoms.

Table S2: Comparison of mass activities with the Ir-Cu mixed oxides reported in the literature.

Sample	Mass Activity vs. RHE [mA mg^{-1}]	Overpotential [mV]	Tafel slope [mV dec^{-1}]
Ir/TiON$_x$/C	626 ± 49	280	58.3 ± 0.3
Ir/CuTiON$_x$/C	840 ± 33	280	58.0 ± 0.9
$Cu_{1.11}Ir$ nanocages[1]	73	280	43.8
$Cu_{0.3}Ir_{0.7}O_{\square}$[2]	50	350	63
$Ir_{0.89}Cu_{0.11}$ HO-np[3]	140	250	52
IrNiCu DNF/C[4]	460	300	/
Co-IrCu ONC/C[5]	640	300	/
TiONx-1h-Ir[6]	360.9	320	60
TiONx-3h-Ir[6]	520	320	/
TiONx-6h-Ir[6]	369.5	320	/
TiO_2(P25)N$_x$-Ir[6]	143.9	320	71
Ir-ND/ATO[7]	69.8	280	56.4
IrNiO$_x$[8]	676	300	/
IrOx[8]	325	300	/

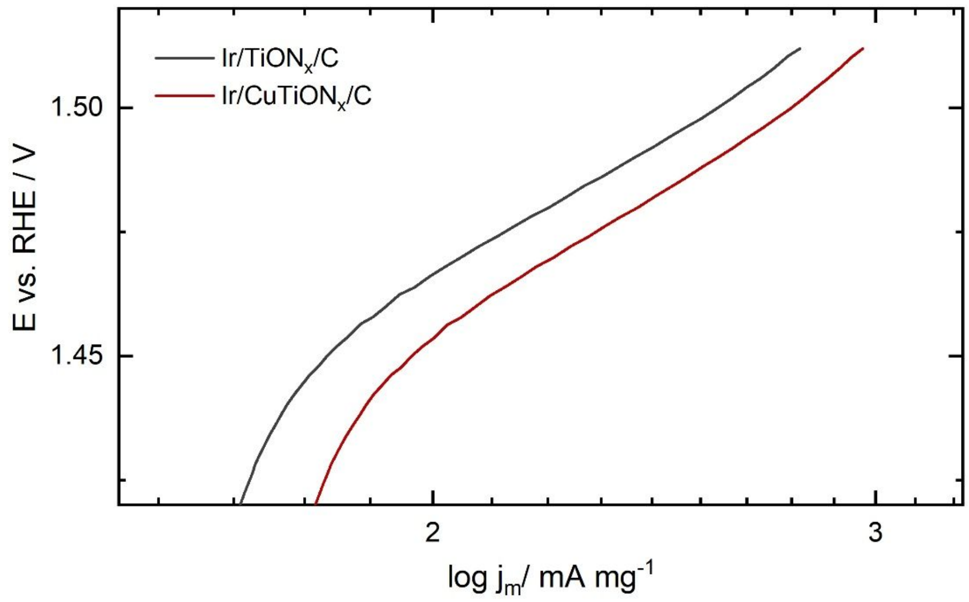

Figure S14: Tafel plots of samples Ir/TiON$_x$/C and Ir/CuTiON$_x$/C.

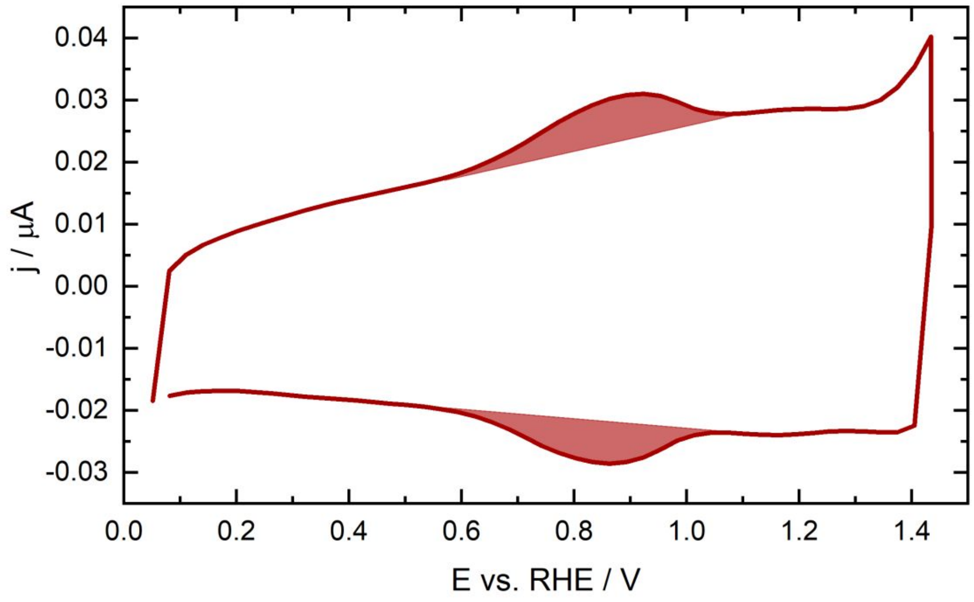

Figure S15: Integrated peak for charge normalization

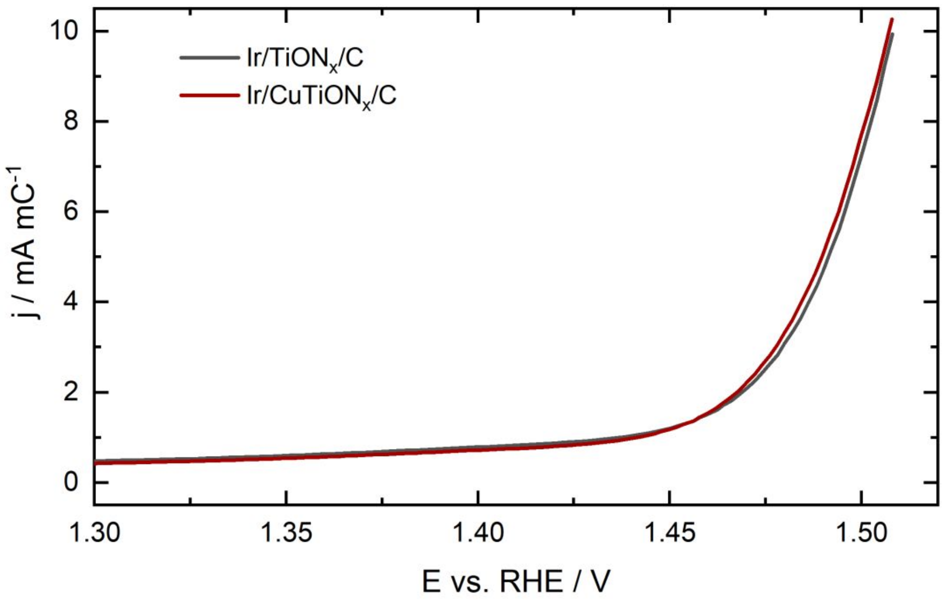

Figure S16: Charge normalized activity of Ir/$TiON_x$/C and Ir/$CuTiON_x$/C.

References:

(1) Wang, C.; Sui, Y.; Xiao, G.; Yang, X.; Wei, Y.; Zou, G.; Zou, B. Synthesis of Cu-Ir Nanocages with Enhanced Electrocatalytic Activity for the Oxygen Evolution Reaction. *J. Mater. Chem. A* **2015**, *3* (39), 19669–19673. https://doi.org/10.1039/c5ta05384f.

(2) Sun, W.; Song, Y.; Gong, X.-Q.; Cao, L.-M.; Yang, J. An Efficiently Tuned D-Orbital Occupation of IrO2 by Doping with Cu for Enhancing the Oxygen Evolution Reaction Activity. *Chem. Sci.* **2015**, *6* (8), 4993–4999. https://doi.org/10.1039/C5SC01251A.

(3) Wang, C.; Moghaddam, R. B.; Bergens, S. H. Active, Simple Iridium−Copper Hydrous Oxide Electrocatalysts for Water Oxidation. *J. Phys. Chem. C* **2017**, *8*, 121. https://doi.org/10.1021/acs.jpcc.6b12164.

(4) Park, J.; Sa, Y. J.; Baik, H.; Kwon, T.; Joo, S. H.; Lee, K. Iridium-Based Multimetallic Nanoframe@Nanoframe Structure: An Efficient and Robust Electrocatalyst toward Oxygen Evolution Reaction. *ACS Nano* **2017**, *11* (6), 5500–5509. https://doi.org/10.1021/acsnano.7b00233.

(5) Kwon, T.; Hwang, H.; Sa, Y. J.; Park, J.; Baik, H.; Joo, S. H.; Lee, K. Cobalt Assisted Synthesis of IrCu Hollow Octahedral Nanocages as Highly Active Electrocatalysts toward Oxygen Evolution Reaction. *Adv. Funct. Mater.* **2017**, *27* (7). https://doi.org/10.1002/adfm.201604688.

(6) Moriau, L.; Bele, M.; Marinko, Ž.; Ruiz-Zepeda, F.; Koderman Podboršek, G.; Šala, M.; Šurca, A. K.; Kovač, J.; Arčon, I.; Jovanovič, P.; Hodnik, N.; Suhadolnik, L. Effect of the Morphology of the High-Surface-Area Support on the Performance of the Oxygen-Evolution Reaction for Iridium Nanoparticles. *ACS Catal.* **2021**, *11* (2), 670–681. https://doi.org/10.1021/acscatal.0c04741.

(7) Oh, H. S.; Nong, H. N.; Reier, T.; Gliech, M.; Strasser, P. Oxide-Supported Ir Nanodendrites with High Activity and Durability for the Oxygen Evolution Reaction in Acid PEM Water Electrolyzers. *Chem. Sci.* **2015**, *6* (6), 3321–3328. https://doi.org/10.1039/c5sc00518c.

(8) Nong, H. N.; Reier, T.; Oh, H. S.; Gliech, M.; Paciok, P.; Vu, T. H. T.; Teschner, D.; Heggen, M.; Petkov, V.; Schlögl, R.; Jones, T.; Strasser, P. A Unique Oxygen Ligand Environment Facilitates Water Oxidation in Hole-Doped IrNiOx Core–Shell Electrocatalysts. *Nat. Catal.* **2018**, *1* (11), 841–851. https://doi.org/10.1038/s41929-018-0153-y.
